# Effects of treated walnut green husk on broiler growth performance, gut health, and meat quality

**DOI:** 10.1016/j.psj.2026.106400

**Published:** 2026-01-06

**Authors:** Hassan Shirzadi, Enayat Rahmatnejad, Shokoufeh Hasanvand, Yaser Khorram Del

**Affiliations:** a Department of Animal Science, Faculty of Agriculture, Ilam University, Ilam, Iran; b Department of Animal Science, Faculty of Agriculture and Natural Resources, Persian Gulf University, Bushehr, 75169, Iran; c Department of Food Analysis and Health, Kifri Technical College, Garmian Polytechnic University, Kifri, KRG, Iraq

**Keywords:** Broiler, Digestibility, Gut health, Meat quality, Performance, Walnut husk

## Abstract

This study evaluated the effects of treated walnut green husk (**WGH**) on growth performance, cecal microflora, jejunal morphology, nutrient digestibility, blood biochemistry, and meat quality in broiler chickens. A total of 180 day-old Ross 308 broilers were assigned to three dietary treatments for a 42-day trial, with six replicates (10 birds per cage). The dietary treatments consisted of a basal diet (control), the basal diet supplemented with 2.1 g/kg fermented WGH (**FWGH**), and the basal diet containing 2.0 g/kg WGH plus 0.1 g/kg multi-enzyme (**EWGH**). Both FWGH and EWGH improved broiler growth performance during the grower, finisher, and overall periods, as indicated by enhanced feed conversion ratio and European production efficiency index, with FWGH producing marginally greater improvements. Both treatments reduced cecal total aerobic bacteria, while FWGH additionally increased *Lactobacillus* spp. and decreased Clostridium perfringens. Jejunal villus height increased in both WGH-treated groups, whereas villus surface area improved in FWGH group. Both FWGH- and EWGH-supplemented diets increased ileal digestibility of dry matter, while FWGH also improved organic matter digestibility. Both FWGH and EWGH had a modulatory effect on blood parameters by increasing lymphocyte (L) percentage and decreasing WBC count, percentage of heterophil (H), H:L ratio, total cholesterol, low-density lipoprotein (LDL), and alkaline phosphatase (ALP) levels. In addition, FWGH increased high-density lipoprotein (HDL) level and reduced alanine transaminase (ALT) activity. Both FWGH and EWGH improved meat quality by reducing breast muscle press loss and malondialdehyde content in thigh muscle, while EWGH further decreased malondialdehyde and increased pH in breast muscle, and enhanced water-holding capacity and pH in thigh muscle compared with the control. Overall, the dietary inclusion of treated-WGH improved growth performance, intestinal health, nutrient digestibility, blood lipid profile, and meat quality in broiler chickens, with FWGH showing slightly superior effects.

## Introduction

Broiler chicken meat is an important food source that provides high-quality protein, relatively low fat content, and essential micronutrients. The global demand for chicken continues to rise, driving the rapid expansion of intensive broiler production systems. However, intensive production systems has been associated with reduced immune function and increased disease susceptibility in broilers (Guerrini et al., 2024). To mitigate these challenges, antibiotics have long been used in poultry production as growth promoters and disease prevention. Nevertheless, the overuse of antibiotics has led to antimicrobial resistance and food safety concerns, prompting global restrictions on their application (Andrew Selaledi, et al., 2020; Wang, et al., 2024; Wu, et al., 2024). Consequently, increasing attention has been directed toward natural feed additives, including herbal extracts, phytochemicals, polyphenols, polysaccharides, probiotics, and as promising alternatives to conventional antibiotics (Alimohammadi, et al., 2024; de Castilho Heiss, et al., 2025; Habibi, et al., 2024; Kuralkar and Kuralkar, 2021; Nazari, et al., 2024; Rahmatnejad, et al., 2024; Wang, et al., 2024; Wickramasuriya, et al., 2024).

Walnut (*Juglans regia*) green husk (WGH) is the primary by-product of walnut processing, constituting around 45–60% of the total weight of fresh walnut fruit (Barekat, et al., 2022). WGH has a history of traditional medicinal use, and walnuts are a rich source of plant secondary metabolites. WGH contains numerous bioactive compounds, including alkanes, alkenes, naphthoquinones, terpenes, sterols, polyphenols and flavonoids, and polysaccharides (Barekat, et al., 2022; Wang, et al., 2023a).

Several phenolic compounds have been identified in WGH, such as hydroxycinnamic acids (chlorogenic acid, caffeic acid, ferulic acid, and sinapic acid), hydroxybenzoic acids (gallic acid, ellagic acid, syringic acid, and vanillic acid), flavonoids (catechin, epicatechin, and myricetin), and juglone (Stampar, et al., 2006). Flavonoids are known for their antioxidant, anti-inflammatory, and anti-allergic activities, as well as their liver protection, antiviral, anticancer, and antithrombotic effects (Michalak, 2022; Pyo, et al., 2024; Ullah, et al., 2020). Some flavonoid-rich plants also exhibit antitumor, antispasmodic, antifungal, and antibacterial properties (Quarenghi, et al., 2000) and are thus considered safe and efficacious in enhancing production in broilers (Prihambodo, et al., 2020).

Walnut leaves and husks has been reported to be rich in polyphenols with antioxidant activity, which may stimulate immune system (Masek, et al., 2019; Stampar, et al., 2006). Additionally, walnut leaf essential oil demonstrates significant antimicrobial activity (Rather, et al., 2012). Several studies have reported the antimicrobial effects of walnut leaves, husks, and their derived extracts (Altemimi, et al., 2023; Pereira, et al., 2008, 2007). Moreover, WGH, owning its antioxidant compounds, including flavonoids and phenolics, may scavenge free radicals and alleviate their detrimental effects on muscle, thus improving meat quality (Oliveira, et al., 2008). Wu, et al. (2024) demonstrated that dietary supplementation with 10.0 g/kg WGH extract improved intestinal health, meat quality, and antioxidant status in broilers, suggesting its potential as a functional additive in poultry production.

Despite its beneficial properties, the plant cell wall limits the release of active compounds in WGH, and the digestive enzymes of poultry are insufficient to fully liberate them. Supplementation with commercial enzymes such as cellulase, hemicellulase, or pectinase can degrade the cell wall and release secondary metabolites such phenolic content (Attia, et al., 2022; Tran, et al., 2025; Wan, et al., 2017). In addition to enhancing the release of secondary metabolites present in WGH, dietary enzyme inclusion confers several other additional benefits. Specifically, enzyme supplementation in broiler diets enhances nutrient digestibility, improves feed conversion ratio, reduces the adverse effects of antinutritional factors, reduces feed costs, improves litter quality, and contributes to environmental protection by decreasing the excretion of organic matter (Attia, et al., 2022; del Alamo, et al., 2008; Giacobbo, et al., 2021).

The short retention time of feed in the poultry digestive tract limits the duration of enzyme activity for effective breakdown of the WGH cell wall. Therefore, applying an enzyme-based pre-fermentation process under laboratory conditions prior to dietary inclusion may enhance the release of secondary metabolites from WGH and improve its biological functionality. Fermentation is a process in which microorganisms such as bacteria, fungi, and yeasts produce enzymes under aerobic or anaerobic conditions, leading to biochemical modifications of feed components. Additionally, fermentation can generate simple compounds such as acetic acid, lactic acid, ethanol, and other organic metabolites (Renge, et al., 2012). In the present study, instead of directly using microorganisms directly for fermentation, their enzymatic products were employed to achieve the benefits of fermentation. Fermentation improves feed palatability, nutrient digestibility, and gut health (Feng, et al., 2007; Niu, et al., 2019; Song, et al., 2008) by breaking plant cell walls, releasing phenolics, and enhancing antioxidant activity (Niu, et al., 2019). Moreover, it increases phytase production, improves phosphorus availability, lowers gut pH, and strengthens immune responses (Feng, et al., 2007; Lim, et al., 2010; Niba, et al., 2009).

Therefore, WGH represents a promising source of natural compounds with antioxidant and antimicrobial activities, suggesting its potential as an alternative to antibiotics. However, its effects on growth performance and physiological responses in broilers have not been fully elucidated. Hence, the present study aimed to investigate the effects of WGH and treated WGH on growth performance, excreta microflora, jejunal morphology, nutrient digestibility, blood biochemical parameters, and meat quality in broilers, thereby providing a scientific basis for the development of antibiotic alternatives in poultry production.

## Materials and methods

All animal care and experimental procedures were conducted in accordance with ethical guidelines and approved by the Research Animal Ethics Committee of Ilam University, Iran (Approval ID: IR.ILAM.REC.1404.021).

### Walnut green husk fermentation

To prepare fermented WGH, 0.1 g of a commercial multi-enzyme was used per 2 g of WGH. Since digestive enzymes require a liquid environment for their catalytic activity, a suspension of the enzyme and WGH was prepared using water. Considering that the catalytic activity of digestive enzymes increases up to 40 °C and declines beyond this temperature (Ravindran, 2013), the suspension was placed in an oven to reach this temperature. As the optimal pH for most digestive enzymes ranges from 4 to 6 (Ravindran, 2013), the suspension pH was initially adjusted to 4 and maintained for 1 h. Thereafter the pH was sequentially adjusted hourly to pH 4.5, 5, 5.5, and finally 6.0. The suspension was then dried at 40 °C and incorporated into the respective experimental diet. Each 2.1 g of the resulting powder contained 2 g of WGH and 0.1 g of the multi-enzyme. The WGH was collected at the nut maturation stage, powdered, and then used. The multi-enzyme used in this study was EndoPower® (Easybio Co., Cheonan, South Korea) containing α-galactosidase, galactomannanase, xylanase, and β-glucanase as primary components, along with amylase, phytase, and protease as secondary components.

### Experimental design, diet, birds’ management, and sampling

A total of 180 day-old male Ross 308 broiler chickens with similar initial body weight were obtained from a commercial hatchery and randomly allocated, in a completely randomized design, to three dietary treatment groups with six replicates per treatment (10 birds per cage). The birds were reared over a 42-day experimental period. The experimental treatments consisted of a control group fed a basal diet without additives, a group fed the basal diet supplemented with 2.1 g/kg fermented walnut green husk (FWGH), and a group fed the basal diet containing 2 g/kg walnut green husk plus 0.1 g/kg multi-enzyme (EWGH). Corn–soybean meal-based basal diets were formulated for the starter (d 1–10), grower (d 11–24), and finisher (d 25–42) phases according to Ross management guidelines. The ingredient composition and chemical analysis of basal diets are presented in Table 1. Feed and water were offered ad libitum throughout the trial. The ambient temperature was set at 32°C on day 1 and gradually decreased to 22°C by the end of the experiment, following commercial broiler management recommendations. On day 1, birds were exposed to 24 h of continuous light. From day 2 until the end of the first week, a lighting schedule of 23 h light and 1 h darkness was applied. Thereafter, a schedule of 20 h light and 4 h darkness was maintained until three days before the end of the trial, when the program was adjusted to 23 h light and 1 h darkness.

**Table 1:** Ingredient and nutrient composition of basal diets during starter (d 0-10), grower (d 11-24), and finisher (d 25-42) phases.

Item	Starter	Grower	Finisher
Ingredients (mg/kg)			
Corn Grain	551.8	599.2	641.5
Soybean Meal	382.1	331.8	285
Soybean Oil	19.4	25.8	33.2
DL- Methionine	3.5	3.10	2.8
L- Lysine HCl	2.5	2.30	2.2
L- Threonine	1.3	1.1	0.9
Vitamin and Mineral Premix^1^	5.0	5.0	5.0
Dicalcium Phosphate	18.5	16.6	14.9
Limestone	9.5	8.8	8.2
Common Salt	1.9	1.9	2.0
Sand^2^	2.1	2.1	2.1
NaHCO_3_	2.4	2.3	2.2
Calculated Analysis (mg/kg, unless stated otherwise)			
ME (Kcal/kg)	2,900	3,000	3,100
Crude Protein	219.5	200.0	181.8
Linoleic Acid	13.67	14.51	15.26
Crude Fiber	38.89	36.41	34.07
SID^3^ Lysine	12.37	11.13	9.98
SID Arginine	13.24	11.91	10.66
SID Threonine	8.31	7.45	6.68
SID Methionine	6.35	5.78	5.29
SID Methionine + Cyst(e)ine	9.18	8.42	7.75
SID Tryptophan	2.31	2.06	1.83
SID Leucine	16.34	15.20	14.14
SID Isoleucine	8.39	7.58	6.83
SID Valine	9.09	8.30	7.57
Calcium	9.28	8.42	7.65
Available Phosphorus	4.64	4.21	3.83
Sodium	1.55	1.55	1.55
Potassium	9.33	8.46	7.65
Chloride	2.01	2.01	2.02
DCAB^4^ (mEq/kg)	249	227	206
Analyzed Values (mg/kg)			
Crude protein	219.1	200.2	181.4
Crude fiber	38.70	36.08	33.90
Total Lysine	13.44	12.26	10.89
Total Arginine	15.12	13.34	11.43
Total Threonine	9.64	8.63	7.77
Total Methionine	3.93	3.54	3.28
Total Methionine + Cyst(e)ine	10.66	9.83	9.01
Total Tryptophan	2.70	2.39	2.11
Total Leucine	19.59	17.88	16.62
Total Isoleucine	10.02	9.07	8.25
Total Valine	10.79	9.85	8.84
Calcium	9.24	8.44	7.60
Total Phosphorus	7.43	6.79	6.36
Sodium	1.55	1.56	1.57
Potassium	9.40	8.38	7.70
Chloride	2.03	2.00	2.04

1 Vitamin and mineral supplement in per kilogram of diet: vitamin A: 9,000 IU;: vitamin D (Cholecalciferol): 2,000 IU; vitamin E: 18 IU; vitamin K: 2 mg; Riboflavin: 6.6 mg; niacin: 30 mg; pantothenic acid: 10 mg; pyridoxine: 3 mg; Folic acid: 1 mg; Thiamine: 1.8 mg; Cyanocobalamin: 15 μg; biotin: 0.1 mg; Choline chloride: 500 mg and Ethoxyquin: 0.1 mg.

2 The basal diets contained 2.1 g/kg of sand, which was replaced either by fermented walnut green husk or by walnut green husk plus the multi-enzyme to formulate the respective diets.

3 Standardized ileal digestible.

4 Dietary cation anion balance.

### Chemical analysis

The nutritional composition of WGH, feed ingredients, and experimental diets was analyzed in accordance with AOAC (2007) guidelines. Analyses included dry matter, crude fat, crude protein, crude fiber, crude ash, calcium, total phosphorus, sodium, potassium, and chlorine. Crude protein was determined by measuring nitrogen content and multiplying it by 6.25. Amino acid profiles were analyzed using an automatic amino acid analyzer (L-8800, Hitachi, Tokyo, Japan) following hydrolysis of the samples in 6 M hydrochloric acid at 110°C for 24 h. Gross energy of WGH and fermented WGH was determined using an adiabatic bomb calorimeter (Parr Instrument Company, Moline, IL), which was calibrated with benzoic acid as the standard.

Additionally, the WGH and fermented WGH samples were analyzed for total antioxidant capacity (TAC), total phenolic content, total flavonoid content, and total flavan content. TAC was determined using the IC₅₀ method based on the scavenging activity of the DPPH (1,1-diphenyl-2-picrylhydrazyl) radical, measured spectrophotometrically (Chem 200, Gesan Production Srl, Campobello, Italy) according to the method described by Brand-Williams (1999). Total phenolic content expressed as mg gallic acid equivalents (GAE)/g dry matter, was determined using the Folin–Ciocalteu method (Singleton, 1999). Total flavonoid content and total flavan were quantified as mg quercetin equivalents (QE)/g dry matter using the aluminum chloride colorimetric assay (Zhishen, et al., 1999) and DMACA (4-dimethylaminocinnamaldehyde) methods (Arnous, et al., 2001), respectively. The chemical composition of WGH is presented in Table 2.

**Table 2:** Chemical composition of WGH1 and fermented WGH.

Item	WGH	Fermented WGH
Ingredients (%, unless otherwise stated)		
Gross Energy, Kcal/kg	4,257	4,163
Dry Matter	90.76	92.05
Organic Matter	86.11	87.47
Crude Protein	3.83	3.92
Ether Extract	0.325	0.305
Crude Ash	4.65	4.58
Crude Fiber	29.82	23.65
Calcium	0.470	0.454
Phosphorus	0.191	0.183
Sodium	0.057	0.058
Potassium	2.490	2.543
Chlorine	0.142	0.142
Phenolic Compounds and Total Anitioxidant Capacity		
Total Phenolics, mg GAE^2^/g	61.37	70.45
Total Flavonoids, mg QE^3^/g	49.85	57.51
Total Flavans, mg QE/g	16.41	18.04
Total Antioxidant Capacity, mg/kg	168	143

^1^Walnut green husk.

^2^Gallic acid equivalent.

^3^Quercetin acid equivalent.

### Growth performance

At the end of each phase (days 10, 24, and 42), pen-wise body weight and feed intake (FI) of each pen were recorded. Mortality was monitored daily throughout the experimental period. Based on these data, body weight gain (**BWG**), feed conversion ratio (**FCR**), and European production efficiency index (**EPEI**) were calculated on a pen basis. The EPEI was calculated using the formula:

$$\text{EPEI},\;\%=100\;\times \left(\frac{\text{livability},\;\%\;\times \;\text{live}\;\text{weight}}{\text{age},\;d\;\times \;\text{FCR}}\right)$$

At the end of the experiment, four birds per pen were randomly selected and humanely euthanized by cervical dislocation for the assessment of cecal microflora, intestinal morphology, nutrient digestibility, and meat quality parameters. For blood biochemistry, three birds per pen were randomly selected, and blood samples were collected from the wing vein using heparinized syringes.

### Cecal microflora

At the end of the experiment, for the collection of fresh cecal samples, the gastrointestinal tract was immediately excised following slaughter. The ceca were opened using a sterile scalpel blade, and the contents were aseptically collected. A 1-g sample of fresh cecal contents from each bird was placed in a sterile Falcon tube containing 9 mL of phosphate-buffered saline (PBS) and ten sterile 3-mm glass beads, followed by vortexing. Serial dilutions (10³ to 10⁷) were subsequently prepared, and the drop-plate method was used for colony enumeration. Briefly, 10 μL of each dilution was inoculated onto predefined spots of agar plates. Total aerobic bacteria, *Lactobacillus* spp., Bifidobacterium spp., *Clostridium perfringens*, and *Campylobacter jejuni* were enumerated using Plate Count Agar, MRS Agar, Bifidobacterium Selective Agar, SPS Agar, and Campylobacter Selective Agar, respectively, following incubation at 37°C for 24 h. Results were expressed as log₁₀ colony-forming units per gram of ileal content (log_10_ CFU/g).

### Jejunal morphology

At the end of the experiment, jejunal tissue samples (approximately 1 cm in length) were collected from slaughtered birds and fixed in 10% neutral-buffered formalin for 24–48 h, following the procedure described by Nazari, et al. (2024). The samples were dehydrated, embedded in paraffin, and sectioned at a thickness of 5 μm using a rotary microtome (Model 3004 M, Germany). The sections were stained with hematoxylin and eosin (H&E) and examined using a light microscope (BX41, Olympus, Tokyo, Japan). Morphological parameters related to intestinal development and absorptive capacity were evaluated, including villus height (VH), crypt depth (CD), villus width (VW), the villus height-to-crypt depth ratio (VH:CD), and villus surface area (VSA). Digital images were captured using a microscope-mounted camera (DP72, Olympus, Tokyo, Japan), and measurements were performed with image analysis software (QWinPlus, version 3.1.0; Leica Cambridge Ltd., UK). For each bird, at least 10 well-oriented villi were measured to ensure accuracy and representativeness.

### Nutrient digestibility

At the end of the experiment, the apparent ileal digestibility (AID) of nutrients was determined using the chromium oxide as an indigestible marker, following the method described by Ghasemi, et al. (2025). On day 37 of the trial, the diets were supplemented with 0.4% chromium oxide for five consecutive days. Ileal digesta were then collected, freeze-dried, and ground using a hammer mill equipped with a 0.5-mm screen. Organic matter and ash concentrations were analyzed in both feed and ileal samples according to AOAC (2007). Chromium oxide concentrations in feed and digesta samples were determined by UV spectrophotometry (Shimadzu UV-1201, Japan) according to the method described by Williams, et al. (1962).

The AID of dry matter was calculated according to Tej, et al. (2025), while the AID of organic matter and ash was determined following Ghasemi, et al. (2025), using the following equations:

$$\text{DMAID},\;\%=1-\left(\frac{\text{Marker}\;\text{in}\;\text{feed},\;\%}{\text{Marker}\;\text{in}\;\text{digesta},\;\%}\right)$$

$$\text{AID},\;\%=1-\left(\;\frac{\text{Marker}\;\text{in}\;\text{feed},\;\%\;}{\text{Marker}\;\text{in}\;\text{digesta},\;\%}\;\times \;\frac{\text{Nutrient}\;\text{in}\;\text{digesta},\;\%}{\text{Nutrient}\;\text{in}\;\text{feed},\;\%}\right)$$

Where, DMAID and AID are apparent digestibility of dry matter and nutrients, respectively.

### Blood biochemistry

Blood samples were collected from the broiler chickens using heparinized syringes, and the samples were immediately divided into two portions after collection. One portion was centrifuged (3,000 rpm for 10 min) to separate plasma. Plasma concentrations of triglycerides, cholesterol, high-density lipoprotein (HDL), low-density lipoprotein (LDL), very low-density lipoproteins (VLDL), total protein, albumin, and globulin, as well as the activities of alkaline phosphatase (ALP), alanine aminotransferase (ALT), and aspartate aminotransferase (AST), were determined using commercial assay kits (Pars Azmoun, Tehran, Iran). In addition, the total antioxidant capacity (TAC) of plasma was measured using a commercial kit (TaliGene Pars Ltd., Isfahan, Iran).

The second portion of blood was collected for hematological analyses. Packed cell volume (PCV) values were measured using Microhematocrit capillary tubes and a microhematocrit centrifuge (12,000 rpm for 10 min). Red blood cell (RBC) and white blood cell (WBC) counts were determined using a Neubauer hemocytometer and a light microscope. Blood smears were also prepared for differential leukocyte counts, including heterophils (H), lymphocytes (L), and eosinophils, and the H:L ratio was subsequently calculated (Nazari, et al., 2024).

### Meat quality

At the end of the experiment, the left breast (pectoralis major) and thigh muscles were evaluated for meat chemical composition, color attributes, and quality parameters. Dry matter, organic matter, crude protein, and ash contents were determined according to AOAC (2007). Meat color indices, including lightness (L*), redness (a*), yellowness (b*), hue angle, and chroma were measured. Color measurements were performed using a portable colorimeter (Lutron, model RGB-1002, Taiwan). According to Ncho, et al. (2025) the hue angle was calculated as tan^−1^(b*/a*), and the chroma index was calculated as (a^⁎2^ + *b*^⁎2^)^0.5^. Additional meat quality parameters, including pH, water-holding capacity (WHC), drip loss, cooking loss, pressing loss, and malondialdehyde (MDA) concentration were also determined according to established methods (Ahammad and Kim, 2024, 2025; Pu, et al., 2025; Sharifi, et al., 2025).

### Statistical analysis

Statistical analysis was performed using the General Linear Model (GLM) procedure in SAS software (SAS Institute, 2008), based on a completely randomized design. The statistical model applied was as follows:

$$Y_{i}=\mu +T_{i}+e_{i}$$

Where, Y_i_ = observation, µ = overall average, T_i_ = effect of i^th^ treatment, e_i_ = error associated with each observation. The Shapiro-Wilk and Levene tests were used to assess the normality of residuals and homogeneity of variances in the data, respectively. Data variability was expressed as the standard error of the mean (SEM). Significant differences among means were analyzed using Tukey’s test at a significance level of *P* < 0.05.

## Results

### Growth performance

As shown in Table 3, both the FWGH and EWGH groups showed lower FCR and higher EPEI compared with the control during the grower, finisher, and overall rearing periods (*P* < 0.05). Birds fed the FWGH diet showed slightly superior performance compared with the EWGH group. FI and BWG were not significantly affected throughout the experimental periods (*P* > 0.05). Nevertheless, across the entire rearing period, BWG of the FWGH and EWGH groups tended to be higher compared with the control (*P* = 0.06).

**Table 3:** Effect of dietary treatments on the growth performance of broiler chickens^1^.

Item	Treatments				
	Control	FWGH	EWGH	SEM	P-value
**Starter (0 to 10 d)**					
Feed intake, g	325	317	315	8	0.65
Body weight gain, g	269	277	264	6	0.37
Feed conversion ratio	1.21	1.15	1.20	0.04	0.47
European production efficiency factor	225	242	221	12	0.43
**Grower (11 to 24 d)**					
Feed intake, g	1229	1178	1197	23	0.34
Body weight gain, g	734	773	754	15	0.23
Feed conversion ratio	1.68^a^	1.53^b^	1.59^ab^	0.03	0.03
European production efficiency factor	314^b^	362^a^	339^ab^	11	0.05
**Finisher (25 to 42 d)**					
Feed intake, g	3218	3129	3150	55	0.51
Body weight gain, g	1360	1441	1408	28	0.18
Feed conversion ratio	2.37^a^	2.17^b^	2.24^b^	0.02	< 0.01
European production efficiency factor	320^b^	369^a^	350^ab^	9	0.01
**Overall (0 to 42 d)**					
Feed intake, g	4772	4624	4662	69	0.34
Body weight gain, g	2363	2491	2425	33	0.06
Feed conversion ratio	2.02^a^	1.86^b^	1.92^b^	0.02	< 0.01
European production efficiency factor	279^b^	320^a^	300^a^	5	< 0.01

^a-b^Means within a row without common superscript are significantly different (*P* < 0.05).

1 Abbreviations: **FWGH**, fermented walnut green husk; **EWGH,** walnut green husk plus multi-enzyme.

### Cecal microflora

The effects of dietary treatments on cecal microflora in broiler chickens are shown in Table 4. Birds in the FWGH and EWGH groups showed reduced total aerobic bacterial counts (*P* < 0.05). In the FWGH group, *Lactobacillus* spp. Counts were higher and *Clostridium perfringens* counts were lower compared with the control (*P* < 0.05). Although EWGH increased *Lactobacillus* spp. and decreased *C. perfringens* populations, these differences were not statistically significant relative to the control (*P* > 0.05). Colonization of *Campylobacter jejuni* and Bifidobacteria was not affected by the dietary treatments (*P* > 0.05).

**Table 4:** Effect of dietary treatments on the cecal microflora of broiler chickens (log_10_ cfu/g)^1^.

Item	Treatments				
	Control	FWGH	EWGH	SEM	P-value
Total aerobic bacteria	8.05^a^	7.57^b^	7.32^b^	0.10	< 0.01
Lactobacilli	7.22^b^	7.60^a^	7.39^ab^	0.05	< 0.01
Bifidobacteria	6.93	7.36	7.51	0.18	0.12
*Clostridium perfringens*	7.04^a^	6.42^b^	6.78^ab^	0.10	< 0.01
*Campylobacter jejuni*	7.66	7.38	7.53	0.09	0.13

^a-b^Means within a row without common superscript are significantly different (*P* < 0.05).

1 Abbreviations: **FWGH**, fermented walnut green husk; **EWGH,** walnut green husk plus multi-enzyme.

### Jejunal morphology

**Fig. 1:**
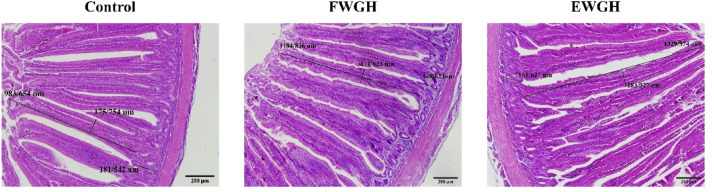
The histological features of the mucosa of jejunum in 42-days-old broiler chickens.

Abbreviations: **FWGH**, fermented walnut green husk; **EWGH,** walnut green husk plus multi-enzyme.

The effects of dietary treatments on jejunal morphology of broiler chickens are shown in Table 5 and Fig. 1. Both FWGH and EWGH groups significantly increased VH compared with the control (*P* < 0.05), with the greatest effect observed in the EWGH group (*P* < 0.05). VSA was also significantly improved by FWGH (*P* < 0.05), whereas the increase observed with EWGH was not statistically significant (*P* > 0.05). VW, CD, and the VH:CD ratio were not affected by the dietary treatments (*P* > 0.05).

**Table 5:** Effect of dietary treatments on the jejunal morphology of broiler chickens^1^.

	Treatments				
Item	Control	FWGH	EWGH	SEM	P-value
Villus height, μm	1062^c^	1207^b^	1355^a^	25	< 0.01
Villus width, μm	98.8	119	99.0	6.0	0.07
Crypt depth, μm	141	146	128	14	0.66
VH:CD	7.65	8.37	11.9	1.26	0.08
VSA, mm^2^	0.332^b^	0.453^a^	0.419^ab^	0.024	0.01

^a-b^Means within a row without common superscript are significantly different (*P* < 0.05).

1 Abbreviations: **FWGH**, fermented walnut green husk; **EWGH,** walnut green husk plus multi-enzyme; **VH:CD**, villus height to crypt depth ratio; **VSA**, villus surface area.

### Nutrient digestibility

The effects of dietary treatments on the ileal nutrient digestibility of broiler chickens are shown in Table 6. Diets containing FWGH and EWGH significantly increased the AID of dry matter compared with the control (*P* < 0.05). Organic matter digestibility was significantly improved by FWGH (*P* < 0.05), whereas the improvement observed with EWGH was not significant relative to the control (*P* > 0.05). Ash digestibility was not affected by the dietary treatments (*P* > 0.05).

**Table 6:** Effect of dietary treatments on the nutrient digestibility coefficients of broiler chickens^1^.

	Treatments				
Item	Control	FWGH	EWGH	SEM	P-value
Dry matter	0.690^b^	0.715^a^	0.707^a^	0.003	< 0.01
Organic matter	0.739^b^	0.770^a^	0.750^ab^	0.007	0.05
Ash	0.580	0.594	0.607	0.007	0.11

^a-b^Means within a row without common superscript are significantly different (*P* < 0.05).

1 Abbreviations: **FWGH**, fermented walnut green husk; **EWGH,** walnut green husk plus multi-enzyme.

### Blood biochemistry

The effects of dietary treatments on blood biochemistry in broiler chickens are shown in Table 7. The inclusion of treated WGH in broiler diets significantly increased plasma TAC. Dietary supplementation with treated WGH significantly reduced plasma total cholesterol, LDL, and ALP levels (*P* < 0.05). Birds receiving FWGH exhibited higher high-density lipoprotein (HDL) concentrations and lower ALT activity (*P* < 0.05). Diets containing FWGH and EWGH increased lymphocytes percentage while reducing WBC counts, heterophil percentage, and the H:L ratio (*P* < 0.05). However, these treatments did not affect PCV, RBC counts, or eosinophil percentage (*P* > 0.05).

**Table 7:** Effect of dietary treatments on the blood biochemistry of broiler chickens^1^.

Item	Treatments				
	Control	FWGH	EWGH	SEM	P-value
TAC, nmol Trolox equivalents/ml	1.77^b^	2.28^a^	2.12^a^	0.09	0.01
Triglyceride, mg/dl	127	98.2	82.6	10.0	0.11
Cholesterol, mg/dl	184^a^	158^b^	146^b^	4	0.02
High-density lipoprotein, mg/dl	56.5^b^	66.9^a^	49.1^b^	1.5	< 0.01
Low-density lipoprotein, mg/dl	92.0^a^	68.5^b^	60.1^b^	2.4	< 0.01
Very low-density lipoproteins, mg/dl	25.4	19.7	16.5	2.01	0.11
Total protein, g/dl	2.77	3.45	3.14	0.16	0.13
Albumin, g/dl	2.09	2.04	2.14	0.16	0.91
Globulin, g/dl	0.680	1.41	0.993	0.178	0.13
Alkaline phosphatase, u/l	284^a^	236^b^	213^b^	4	< 0.01
Alanine transaminase, u/l	323^a^	245^b^	315^a^	4	< 0.01
Aspartate transaminase, u/l	5.75	3.14	2.10	0.91	0.13
Packed cell volume, %	26.6	28.2	29.3	0.7	0.10
Red blood cells, × 10^6^/µl	2.25	2.61	2.46	0.01	0.11
White blood cells, × 10^3^/µl)	9.49^a^	8.52^b^	8.15^b^	0.09	< 0.01
Heterophil, %	27.5^a^	22.1^b^	20.1^b^	1.0	< 0.01
Lymphocyte, %	68.9^b^	75.5^a^	77.4^a^	1.1	< 0.01
Eosinophil, %	3.57	2.49	2.49	0.30	0.07
Heterophil:lymphocyte ratio	0.400^a^	0.292^b^	0.259^b^	0.020	< 0.01

^a-b^Means within a row without common superscript are significantly different (*P* < 0.05).

1 Abbreviations: **FWGH**, fermented walnut green husk; **EWGH,** walnut green husk plus multi-enzyme; **TAC**, total antioxidant capacity.

### Meat quality

The effects of the dietary treatments on meat chemical composition and quality in broiler chickens are presented in Table 8, Table 9, respectively. The treatments did not affect the dry matter, organic matter, crude protein, or ash content of breast and thigh muscles (*P* > 0.05). Both FWGH and EWGH diets reduced breast muscle press loss and MDA levels in thigh muscle (*P* < 0.05). Feeding EWGH also decreased MDA and increased pH in breast muscle, and increased WHC and pH in thigh muscle compared with the control (*P* < 0.05), whereas FWGH improved these parameters without producing significant differences relative to the control (*P* > 0.05). Additionally, the dietary treatments did not affect meat color parameters, including lightness, redness, yellowness, hue angle, and chroma of breast and thigh muscles (*P* > 0.05).

**Table 8:** Effect of dietary treatments on the meat chemical composition of broiler chickens (%)^1^.

Item	Treatments				
	Control	FWGH	EWGH	SEM	P-value
**Breast**					
Dry matter	26.4	27.1	26.6	0.5	0.65
Organic matter	25.1	25.8	25.5	0.4	0.60
Crude protein	21.6	23.1	22.2	0.8	0.47
Ash	1.24	1.27	1.15	0.10	0.74
**Thigh**					
Dry matter	26.4	27.0	25.5	0.5	0.25
Organic matter	25.0	25.7	24.2	0.5	0.21
Crude protein	20.6	21.8	20.6	0.8	0.49
Ash	1.40	1.26	1.28	0.11	0.60

^a-b^Means within a row without common superscript are significantly different (*P* < 0.05).

1 Abbreviations: **FWGH**, fermented walnut green husk; **EWGH,** walnut green husk plus multi-enzyme.

**Table 9:** Effect of dietary treatments on the meat quality of broiler chickens^1^.

Item	Treatments				
	Control	FWGH	EWGH	SEM	P-value
**Breast**					
pH	7.13^b^	7.43^ab^	7.47^a^	0.07	0.03
Water holding capacity, %	50.0	49.9	49.4	3.0	0.99
Drip loss, %	15.1	13.5	14.3	1.6	0.78
Cooking loss, %	28.9	26.8	27.7	2.1	0.80
Press loss, %	53.2^a^	44.2^b^	45.2^b^	2.1	0.04
Malondialdehyde (nmol/g)	48.6^a^	35.8^ab^	30.1^b^	3.7	0.03
Lightness	135	128	122	10	0.68
Redness	14.0	15.1	18.7	1.7	0.21
Yellowness	22.0	19.3	21.8	1.8	0.53
Hue angle	57.1	51.8	49.8	2.5	0.19
Chroma index	26.1	24.5	28.8	2.2	0.43
**Thigh**					
pH	7.03^b^	7.37^ab^	7.43^a^	0.08	0.03
Water holding capacity, %	45.6^b^	52.4^ab^	57.7^a^	1.9	0.01
Drip loss, %	12.3	11.3	11.2	0.3	0.07
Cooking loss, %	24.3	20.1	19.1	2.4	0.34
Press loss, %	41.5	43.4	42.4	3.4	0.92
Malondialdehyde (nmol/g)	55.3^a^	38.9^b^	38.7^b^	2.6	< 0.01
Lightness	103	114	95.2	10.0	0.47
Redness	18.5	22.2	21.0	1.8	0.40
Yellowness	15.8	18.8	16.9	2.0	0.59
Hue angle	39.8	40.4	38.6	4.2	0.96
Chroma index	24.4	29.1	27.2	1.9	0.30

^a-b^Means within a row without common superscript are significantly different (*P* < 0.05).

1 Abbreviations: **FWGH**, fermented walnut green husk; **EWGH,** walnut green husk plus multi-enzyme.

## Discussion

The improved growth performance observed in broilers fed WGH diets, particularly those receiving fermented WGH, may be linked to enhanced intestinal health. This improvement may be attributed to a reduction in harmful bacteria such as *Clostridium perfringens*, an increase in beneficial *Lactobacillus* populations, increased VH and VSA, and improved digestibility of dry matter and organic matter, all of which contribute to more efficient nutrient utilization and consequently, improved growth performance.

The positive effects of medicinal plants on the growth performance of broiler chickens have been attributed to their antimicrobial compounds, which reduce the colonization of pathogenic bacteria in the gastrointestinal tract and thereby improve nutrient utilization and digestibility. In addition, medicinal plants can enhance bile secretion and stimulate digestive enzyme activity, leading to more efficient nutrient digestion and improved feed efficiency (Ramakrishna Rao, et al., 2003; Windisch, et al., 2008). Medicinal plants may also increase nutrient absorption by promoting villus development in the intestinal mucosa (Lee, et al., 2003). Taken together, the enhanced performance of broilers fed WGH, compared with the control group, appears to result from the markedly higher antioxidant capacity and abundant phenolic constituents present in WGH, as demonstrated by the chemical analysis conducted in this study. As shown in Table 7, the significant increase in plasma total antioxidant capacity (TAC) observed in broilers fed WGH-containing diets further supports the role of WGH as a potent dietary antioxidant source.

The beneficial effect of enzyme supplementation in poultry diets has been well demonstrated, demonstrating improvements in performance through enhanced gut microbiota balance, intestinal barrier function, and nutrient digestibility (Attia, et al., 2020; Gitoee, et al., 2015; Hashim, et al., 2023; Javaid, et al., 2022; Yi, et al., 2024).

Consistent with the present study, several reports have demonstrated beneficial effects of natural feed additives, such as polyphenols, on broiler performance (Aengwanich, et al., 2009; Ahammad and Kim, 2024; de Castilho Heiss, et al., 2025; El-Hack, et al., 2020). However, contrary to our findings, Wu, et al. (2024) found that adding 10.0 g/kg of WGH extract to the diet had no significant impact on body weight, BWG, FI, or FCR. This discrepancy may be attributed to the different forms of walnut husk used (extract versus whole husk) and the potential synergistic effects of combining walnut husk with enzyme supplementation in the present study. The enzyme not only hydrolyzes non-starch polysaccharides, thereby releasing nutrients trapped within plant cell walls, but also breaks down the fibrous structure of WGH (Giovannoni, et al., 2020; Liu, et al., 2023; Niu, et al., 2022; Wan, et al., 2017), facilitating the release of its active compounds with antimicrobial and antioxidant properties in the gut. The slightly superior performance in the FWGH group compared with EWGH may be due to a greater release of bioactive compounds, as the WGH in this treatment was exposed to the enzyme for a longer duration (5 h during pre-fermentation).

The intestinal microbiota plays a crucial role as a protective barrier in the body. However, various factors, particularly diet, can disrupt its homeostasis. Supplementation diets with WGH extracts or WGH-derived polysaccharides has been shown to decrease the abundance of potentially harmful bacteria while simultaneously promoting the growth of beneficial microbial populations (Fang, et al., 2022; Wang, et al., 2021; Wu, et al., 2024; Yang, et al., 2023). Consistent with findings of present study, Wang, et al. (2025) also reported that dietary supplementation with 0.1% WGH extract significantly increased the relative abundance of beneficial bacteria (e.g., *Firmicutes, Lactobacillus*) and reduced pathogenic bacteria (e.g., *Proteobacteria, Shigella*) in pigs. The positive effect of WGH observed in the present study may related to polysaccharides in WGH selectively promoting the colonization of beneficial bacterial content (Jahanban-Esfahlan, et al., 2019). Bioactive components in WGH, such as polyphenols and flavonoids, may exert selective antimicrobial effects against pathogenic bacteria, indirectly enhancing the relative abundance of beneficial bacteria (Wang, et al., 2023b). Polyphenolic compounds exhibit antibacterial activity through several mechanisms, including binding to bacterial proteins (such as adhesins, enzymes, and cell envelope transport proteins), disrupting porin function in Gram-negative bacteria, inhibiting efflux pumps, and blocking enzymes involved in cell wall, membrane fatty acid, and ATP synthesis (Makarewicz, et al., 2021).

In line with our results, Wu, et al. (2024) observed significant improvements in jejunal VH following dietary inclusion of WGH extract in broilers. Additionally, Wang, et al. (2021) demonstrated that WGH extract supplementation in a high-fat diet enhanced antioxidant enzyme activity and increased the expression of intestinal tight-junction proteins. Likewise, Prihambodo, et al. (2020) reported that dietary polyphenol supplementation increased intestinal VH and improved the VH:CD ratio in broilers. The inclusion of WGH in the present study led to significant improvements in intestinal morphology, likely due to its high polyphenol content and the beneficial modulation of gut microbial populations.

The improved AID of dry matter in diets containing FWGH and EWGH, as well as the enhanced AID of organic matter, may be attributed to improvements in intestinal barrier function and beneficial alterations in gut microbial populations induced by WGH, as supported by our findings, together with the synergistic effects of WGH and enzyme supplementation. Wang, et al. (2025) highlighted the physiological significance of increasing beneficial bacteria, such as *Lactobacillus*. As a core intestinal bacterium, *Lactobacillus* promotes digestion and nutrient absorption by breaking down compounds such as carbohydrates. Additionally, it can stimulate intestinal epithelial cells to secrete antimicrobial peptides, thereby strengthening the integrity of the intestinal mucosal barrier. Our findings differ from those of Wu, et al. (2024), who reported that supplementation with WGH extract did not significantly affect dry matter or organic matter digestibility. In the present study, the addition of enzymes likely facilitated the degradation of WGH fiber components, thereby improving the release and absorption of its bioactive compounds and resulting in enhanced nutrient digestibility.

In accordance with our results, dietary supplementation with walnut leaf extract has been reported to decreases serum LDL, blood glucose, and triglyceride levels, while increasing HDL levels in rats (Divband, et al., 2010). In addition, Wang, et al. (2019), investigating the effects of WGH extract on the lipid profile of rats made obese with a high-fat diet, reported that the extract reduced serum triglycerides, total cholesterol, and LDL concentrations. Studies indicate that flavonoids lower plasma LDL concentrations by upregulating LDL receptor (LDLR) expression, although the underlying mechanism remains poorly understood (Bjune, et al., 2024). The cholesterol-lowering potential of WGH is attributed to its major bioactive compounds, such as ellagic acid and flavonoids (Stampar, et al., 2006). Moreover, caffeic acid, one of the principal polyphenols in WGH, has been shown to suppress lipogenic gene expression, decrease cholesterol and triglyceride synthesis, and enhance the phosphorylation of key enzymes regulating lipid metabolism (Liao, et al., 2014). Increased plasma concentrations of hepatic enzymes, alkaline phosphatase and aspartate aminotransferase, may result from hepatocyte membrane damage caused by free-radical invasion or inflammation, leading to leakage of these enzymes into plasma. Therefore, the reduction in hepatic enzyme levels observed in WGH-fed birds, together with the significant increase in plasma TAC, can be attributed to the antioxidant and anti-inflammatory properties of juglone (Ahmad and Suzuki, 2019), gallic acid (Kahkeshani, et al., 2019), ellagic acid (Sharifi-Rad, et al., 2022), and sinapic acid (Chen, 2016) present in WGH. Moreover, ellagic acid has been reported to possess hepatoprotective properties (Sharifi-Rad, et al., 2022), and thus may protect liver cells and prevent the leakage of intracellular enzymes into the bloodstream. Plant polyphenolic compounds act as reducing agents, metal chelators, and free-radical scavengers, thereby neutralizing oxidative stress (Lim, et al., 2010).

Phenolic compounds in plants can prevent the oxidation of meat macromolecules and improve meat quality due to their antioxidant properties, enhanced reducing capacity, and inhibition of free-radical formation (Bai, et al., 2025). It has been reported that Juglone as a bioactive components in WGH may directly inhibit oxidative reactions by binding to reactive oxygen species (ROS), and indirectly by inhibiting ROS-producing enzymes, chelating transition metal ions, donating hydrogen atoms, regenerating vitamin E, and enhancing superoxide dismutase activity, thereby suppressing oxidative stress (Ahmad and Suzuki, 2019).

MDA is a final product of unsaturated fatty acid peroxidation in cells and is produced in considerable amounts in response to elevated free radicals. It is widely regarded as an indicator of oxidative stress and antioxidant status. Therefore, the reduced MDA concentration in breast and thigh muscles in this study can be attributed to the high antioxidant capacity and phenolic content of WGH, as supported by the chemical analysis of the samples in our study. In addition, intestinal inflammation induced by pathogenic bacteria can disrupt tight junctions between epithelial cells, allowing microbial invasion into the bloodstream and muscle tissues. This can lead to bacterial spoilage and reduced meat shelf-life, primarily through bacterial lipid oxidation in muscles and damage to muscle myofibrils, ultimately resulting in low WHC (Insausti, et al., 2001). The improved meat WHC observed in the WGH-fed treatments may be attributed to the antioxidant properties of WGH phenolic compounds of WGH (Oliveira, et al., 2008) as well as the antimicrobial effects (Wang, et al., 2023b). Our results differ from those reported by Wu, et al. (2024), who observed no significant effects of dietary WGH extract on overall meat quality. However, both studies similarly reported no significant effects of walnut husk on meat color, drip loss, or cooking loss. An important aspect of the present study is the potential synergy between walnut husk and enzyme supplementation (FWGH and EWGH). The inclusion of enzymes may have promoted greater degradation of WGH components, thereby increasing the accessibility of its bioactive molecules within the gastrointestinal tract.

## Conclusion

In conclusion, this study highlights the potential of treated WGH, particularly its fermented form, as a functional feed additive in broiler production. Rather than merely improving performance and health parameters, these additives may contribute to more efficient and sustainable poultry systems by enhancing gut function, nutrient utilization, and oxidative stability. The use of treated WGH provides a practical, natural alternative to conventional performance promoters, with FWGH exhibiting slightly superior effects, thereby potentially enhancing overall productivity and meat quality. Further research is needed to investigate varying inclusion levels to better validate and maximize the benefits of treated WGH in broiler chicken diets.

## CRediT authorship contribution statement

**Hassan Shirzadi:** Writing – review & editing, Writing – original draft, Validation, Supervision, Software, Resources, Funding acquisition, Conceptualization. **Enayat Rahmatnejad:** Writing – review & editing, Writing – original draft, Supervision, Investigation, Data curation, Conceptualization. **Shokoufeh Hasanvand:** Writing – review & editing, Validation, Project administration, Methodology, Investigation, Data curation. **Yaser Khorram Del:** Writing – review & editing, Resources, Methodology, Investigation, Formal analysis, Data curation.

## Disclosures

There are no potential conflict of interest regarding this manuscript.
